# Linperlisib causing high blood sugar and hyponatremia, leading to facial nerve paralysis and muscle nerve damage: A rare case report

**DOI:** 10.1097/MD.0000000000042875

**Published:** 2025-06-13

**Authors:** Yu-cai Jiang, Cheng-fei Zhao, Lin-lin Zheng

**Affiliations:** a Department of Pharmacy, Affiliated Hospital of Putian University, Putian, Fujian Province, China; b School of Pharmacy and Medical Technology, Putian University, Putian, Fujian Province, China; c Department of Oncology, Affiliated Hospital of Putian University, Putian, Fujian Province, China.

**Keywords:** facial palsy, hyperglycemia, hyponatremia, linperlisib, myalgia

## Abstract

**Rationale:**

Linperlisib is a highly selective small-molecule inhibitor of phosphatidylinositol-3-kinase delta for the treatment of relapsed/refractory follicular lymphoma.

**Patient concerns:**

The patient was a 64-year-old male with peripheral T-cell lymphoma and diabetes mellitus who experienced hyperglycemia, hyponatremia, facial palsy, and myalgia during linperlisib treatment.

**Diagnoses:**

Drug-induced high blood sugar and hyponatremia.

**Interventions:**

Active management of hyperglycemia during hospitalization and hyponatremia was corrected with active symptomatic treatment.

**Outcomes:**

During hospitalization, the patient’s hyperglycemia and hyponatremia were effectively managed through active symptomatic treatment. However, following a reduction in the linperlisib dose, the patient developed recurrent hyperglycemia, hyponatremia, and new-onset myalgia. Consequently, the patient declined further linperlisib therapy and permanently discontinued the medication.

**Lessons:**

Clinicians should be aware of the risk of complications such as hyperglycemia and hyponatremia in patients with lymphoma and diabetes receiving linperlisib.

## 1. Introduction

Linperlisib is a novel 1st-class drug that was developed independently in China. It is a highly selective small-molecule inhibitor targeting phosphatidylinositol-3-kinase delta (PI3Kδ) and has demonstrated favorable efficacy and safety in the treatment of recurrent and/or refractory follicular lymphoma (FL).^[1]^ The National Medical Products Administration of China granted linperlisib breakthrough therapy designation for the management of recurrent/refractory FL. On November 9, 2022, it was approved for marketing to adult patients with recurrent/refractory FL who had previously undergone at least 2 systemic treatments. The recommended dose is 80 mg once daily until disease progression or intolerable adverse reactions occur.

Furthermore, linperlisib has been investigated in clinical trials for peripheral T-cell lymphoma^[2,3]^ and diffuse large B-cell lymphoma^[4]^ and has demonstrated promising efficacy. Adverse reactions associated with linperlisib include infectious pneumonia, viral reactivation, interstitial lung disease, diarrhea, colitis, adverse hepatic reactions, adverse hematologic reactions, and cutaneous toxicity. Linperlisib has not previously been reported to cause adverse reactions such as facial palsy, hyponatremia, and muscle neuropathy due to poor glucose control. We report a case of linperlisib-induced high blood sugar causing clinical manifestations of facial palsy, hyponatremia, and muscle neuropathy. The patient presented with left-sided drooping of the mouth corner and incomplete eyelid closure accompanied by general symptoms of muscle aches, stiffness, and discomfort.

The patient provided informed consent for publication of this report.

## 2. Case presentation

**Figure 1.**
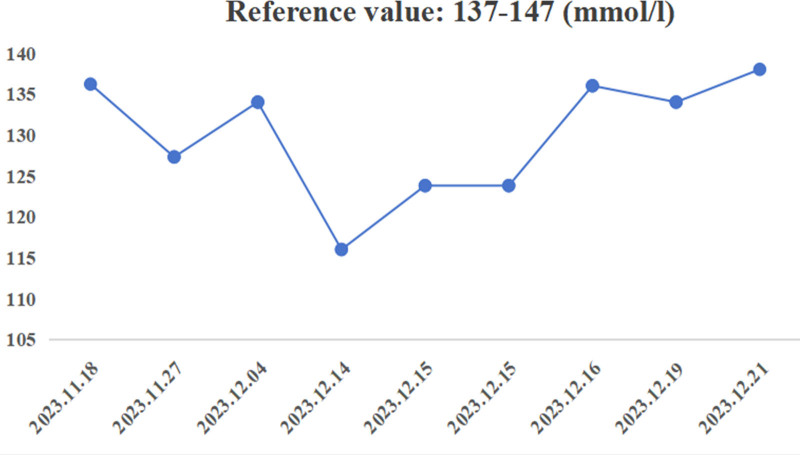
Changes in the patient’s sodium level over time.

A 64-year-old male patient was diagnosed with peripheral T-cell lymphoma 4 years prior to presentation. After multiple lines of treatment, the patient commenced linperlisib 80 mg orally once daily on November 19, 2023. Seven days later, on November 26, 2023, the patient experienced general muscle aches and weakness in their limbs, a left eyelid droop, and a crooked mouth on one side. The patient was subsequently hospitalized on November 27, 2023. The patient had a history of hypertension and diabetes and was concurrently prescribed nifedipine controlled-release tablets (30 mg) and valsartan (80 mg) for hypertension, and Lantus 14 International Unit (IU) and Novolog 16 IU for diabetes. The patient’s blood pressure and sugar levels were well controlled, and neither the patient nor her family had any history of cancer. Clinically, the patient reported general muscle aches, weakness, and pain in the left ear. The patient presented with left eyelid drooping, a slanting mouth, a centrally positioned tongue, muscle strength of level V in all limbs, and a soft neck. The patient’s vital signs were as follows: blood pressure, 111/87 mm Hg; heart rate, 81 beats/min; respiratory rate, 20 breaths/min; and temperature, 36.5°C. The patient had good oxygen saturation (98% in ambient air), weighed 72 kg, and was 170 cm tall. The following biological results were found: elevated blood sugar level of 22.6 mmol/L (3.8–6.38 mmol/L), sodium level of 127.3 mmol/L (137–147 mmol/L; Fig. 1), chloride level of 92.4 mmol/L (99–110 mmol/L), acid pH of 7.360 (7.350–7.450), plasma osmolarity of 284.2 mOsml/L (280–320 mOsml/L), potassium level of 4.29 mmol/L (3.5–5.3 mmol/L), and ionic gap of 11.1 mmol/L (5–16 mmol/L). No abnormalities were found on computed tomography. The patient was diagnosed with peripheral facial palsy and hyponatremia. Facial palsy (facial paralysis) was diagnosed as diabetic facial palsy and was closely associated with poor glycemic control. If glycemic control is poor, diabetic facial palsy can become more severe. Treatments for diabetic facial palsy include the control of blood sugar levels, acupuncture therapy, and nutritional nerve support. The patient presented with general aches, stiffness, weakness, and pain in the posterior left ear, which was considered to be caused by hyponatremia. According to the common terminology criteria for adverse events version 5.0, this was rated as a grade 3 adverse reaction. The patient received sodium replacement therapy and analgesics. They received physical saline (0.25 L for the 1st 2 hours), and their maximum numeric rating scale pain score was 5, which was treated with morphine injection (10 mg intramuscular injection). Acetaminophen hydrocodone (0.33 g once every 6 hours) was also administered. The patient’s condition improved after the treatment.

Based on a rigorous assessment of the linperlisib prescription guidelines, the patient’s clinical condition, and risk-benefit analysis. On December 9, 2024, linperlisib tablets (40 mg once a day) were resumed. On the 4th day after medication administration, the patient’s blood sugar control was poor, and active insulin therapy was initiated. On the 5th day, the patient reported that the body aches, soreness, and fatigue were more severe than before and that they subsequently stopped taking linperlisib. Biologically, the following were found: elevated blood sugar level of 18.4 mmol/L (3.8–6.38 mmol/L),sodium level of 116.0 mmol/L (137–147 mmol/L), chloride level of 82.0 mmol/L (99–110 mmol/L), acid pH of 7.320 (7.350–7.450), plasma osmolarity of 270 mOsml/L (280–320 mOsml/L), potassium level of 5.97 mmol/L (3.5–5.3 mmol/L), ionic gap of 21.5 mmol/L (5–16 mmol/L), calcium level of 1.8 mmol/L (2.03–2.54 mmol/L), HCO3^-^ level of 16.5 mmol/L (21.4–27.3 mmol/L). The patient’s urine glucose level was 1+, and urine ketone levels were normal. Electromyography and nerve conduction velocity testing of the patient revealed peripheral nerve damage in the upper and lower extremities (motor and sensory fiber involvement). The patient presented with general aches, stiffness, weakness, and pain in the posterior region of the left ear, which was considered to be caused by hyponatremia. The common terminology criteria for adverse events terminology criteria for grade 4 adverse events version 5.0 rated this event as a grade 4 adverse reaction. The patient was treated with fluid and sodium replacement, as well as analgesia. carbonic acid (125 mL) and normal saline (0.25 L + 30 mL 10% sodium chloride injection for the 1st 2 hours) were administered. The maximum numeric rating scale pain score was 6, and the patient was administered morphine (10 mg intramuscularly) and oxycodone extended-release tablets (20 mg once every 12 hours). As the patient did not undergo a blood ketone test, we suspected diabetic ketoacidosis with electrolyte metabolic disturbances (hyperkalemia, hyponatremia, and hypochlorhydria).

Based on the patient’s description of their symptoms, we concluded that linperlisib caused poor blood sugar control, resulting in facial paralysis and diabetic ketoacidosis and subsequently triggering hyponatremia. Hyponatremia causes patients to experience generalized muscle aches, swelling, and fatigue. The Naranjo adverse drug reactions probability scale is widely used to evaluate the association between drugs and adverse drug reactions. Naranjo adverse drug reactions probability scale involves 10 questions with the answer choices “yes,” “no,” or “unknown or nonapplicable.” The adverse drug reaction is assigned to a probability category based on a total score of ≥9 as “definite,” 5 to 8 as “likely,” 1 to 4 as “possible,” and 0 as “unlikely.” The results are presented in Table 1. The total score in this case was 9, and the causality was assessed as “definite.”

**Table 1:** Naranjo’s assessment scale for adverse drug reactions.

Axis	Numerical score		
	Yes	No	Unknown
Previous reports on the reaction		0	
The reaction occurred after the use of linperlisib	2		
Sodium increased after linperlisib withdrawal	1		
Linperlisib rechallenge	2		
Exclusion of alternative causes of the reaction		2	
Placebo response		1	
Drug concentration and monitoring			0
Dose relationship			0
Previous exposure and cross-reactivity			0
Presence of any objective evidence	1		
Results	9 = “definite”		

## 3. Discussion

The phosphatidylinositol-3-kinase (PI3K) family of proteins plays a crucial role in cell signaling, particularly in glucose regulation. PI3K consists of regulatory and catalytic subunits, with the latter including different isoforms, such as PI3Kα, PI3Kβ, and PI3Kδ. PI3Kα is a major member of the PI3K family, and its signaling pathway is crucial for growth and metabolism. Studies have shown that targeting PI3Kα disrupts glucose homeostasis.^[5]^ For example, the less selective PI3Kα inhibitor alpelisib has caused severe hyperglycemia side effects in clinical trials, with 51% to 65% of patients experiencing hyperglycemia when taking the PI3Kα inhibitor alpelisib and endocrine therapy, making it the most common grade 3 to 4 adverse reaction leading to drug reduction or discontinuation. Additionally, because PI3Kα plays an important role in maintaining blood sugar stability, its inhibitors may cause severe complications such as hyperglycemia and hyperinsulinemia by indiscriminately affecting both the tumor and normal tissues.^[6]^

Linperlisib targets the PI3Kδ subunit, blocking the B-cell receptor signaling pathway and thus suppressing tumor growth. Linperlisib also modifies the immune microenvironment by downregulating regulatory T cells.^[7]^ PI3Kδ is another important member of the PI3K family, and its inhibitor, linperlisib, has been developed as a highly selective and specific drug intended to reduce the occurrence of adverse effects such as high blood sugar. This suggests that targeting PI3Kδ inhibition may help to regulate blood sugar levels more precisely and avoid the widespread metabolic side effects associated with PI3Kα inhibitors. In a key Phase II clinical trial of linperlisib treatment for FL, of the 84 patients who received treatment, 11 (13.1%) experienced hyperglycemia, and only 1 (1.2%) experienced a grade ≥3 event.^[8]^ Therefore, the adverse effects of linperlisib-induced hyperglycemia should not be underestimated, and it is important to monitor the blood sugar levels in patients taking linperlisib dynamically. It may be helpful to recommend that patients with diabetes follow the standard dietary guidelines for diabetes when starting linperlisib therapy. The management recommendations for hyperglycemia are as follows: Metformin 500 mg/dose, once daily; if there is no gastrointestinal intolerance, the dose can be titrated every 7 days to increase to 500 mg/dose, twice daily, and then increased by 500 mg as needed until the maximum dose of 2000 mg daily; If the maximum dose of metformin cannot control blood sugar, an endocrinologist should be consulted to determine whether insulin sensitizers, such as pioglitazone and/or insulin, should be administered; When a patient’s fasting blood sugar is >13.89 mmol/L, intravenous fluid replacement, correction of electrolytes, and discontinuation of medication are recommended until blood sugar improves to prevent diabetic ketoacidosis; When a patient’s fasting blood sugar is >27.78 mmol/L, the above measures should be used in combination with insulin therapy. If blood sugar levels do not improve within 24 hours, the medication should be discontinued permanently.

## 4. Patient perspective

During the follow-up, the patient initially experienced considerable anxiety regarding his symptoms, indicating disease progression. This led to a loss of hope and even the contemplation of discontinuing treatment. However, with continued symptomatic management by the doctor and detailed medication explanations provided by the pharmacist, the patient realized that these symptoms were severe adverse reactions caused by the drug. After symptom alleviation, the doctors recommended medication resumption; however, the patient initially declined. With guidance from healthcare professionals and thorough explanations of the potential benefits and drawbacks from doctors and clinical pharmacists, the patient carefully weighed the pros and cons before deciding to give it another try. Unfortunately, the adverse reactions recurred, leading to permanent discontinuation of this particular drug and switching to alternative medications. He expresses gratitude to the medical team for attentive monitoring and care throughout his treatment.

## 5. Conclusions

In conclusion, clinicians should be aware that linperlisib treatment in patients with lymphoma carries a risk of developing hyperglycemia, which can lead to facial nerve paralysis, hyponatremia, and muscle neuropathy. Therefore, close monitoring of patients is necessary to detect potential complications early and to avoid life-threatening complications.

## Acknowledgments

The authors appreciate the patient and the guardians of the patient.

## Author contributions

**Conceptualization:** Yu-cai Jiang.

**Data curation:** Yu-cai Jiang.

**Investigation:** Yu-cai Jiang, Lin-lin Zheng.

**Writing – original draft:** Yu-cai Jiang, Cheng-fei Zhao.

**Writing – review & editing:** Yu-cai Jiang, Cheng-fei Zhao, Lin-lin Zheng.
